# A Daytime Nap Does Not Enhance the Retention of a First-Order or Second-Order Motor Sequence

**DOI:** 10.3389/fnbeh.2021.659281

**Published:** 2021-07-16

**Authors:** Michael P. Barham, Jarrad A. G. Lum, Russell Conduit, Lara Fernadez, Peter G. Enticott, Gillian M. Clark

**Affiliations:** ^1^Cognitive Neuroscience Unit, School of Psychology, Deakin University, Geelong, VIC, Australia; ^2^School of Health and Biomedical Sciences, Royal Melbourne Institute of Technology, Melbourne, VIC, Australia

**Keywords:** sleep, statistical/sequence learning, memory, serial reaction time task, first-order and second-order conditional sequences

## Abstract

This study examined the effects of a daytime nap on the retention of implicitly learnt “first-order conditional” (FOC) and “second-order conditional” (SOC) motor sequences. The implicit learning and retention of a motor sequence has been linked to the neural processes undertaken by the basal ganglia and primary motor cortex (i.e., procedural memory system). There is evidence, however, suggesting that SOC learning may further rely on the hippocampus-supported declarative memory system. Sleep appears to benefit the retention of information processed by the declarative memory system, but not the procedural memory system. Thus, it was hypothesized that sleep would benefit the retention of a SOC motor sequence but not a FOC sequence. The implicit learning and retention of these sequences was examined using the Serial Reaction Time Task. In this study, healthy adults implicitly learnt either a FOC (*n* = 20) or a SOC sequence (*n* = 20). Retention of both sequences was assessed following a daytime nap and period of wakefulness. Sleep was not found to improve the retention of the SOC sequence. There were no significant differences in the retention of a FOC or a SOC sequence following a nap or period of wakefulness. The study questions whether the declarative memory system is involved in the retention of implicitly learnt SOC sequences.

## Introduction

The ability to implicitly or incidentally learn and then retain sequentially structured information has been proposed to support motor, social, reading, and language skills (Karni, 1996; Clegg et al., 1998; Van Overwalle, 2009; Hamrick et al., 2018; Hedenius et al., 2020). To date, most research undertaken in this area has focused on the cognitive and neural processes that underpin the learning of sequences (Schwarb and Schumacher, 2012; Janacsek et al., 2020). Less is known about the processes which allow this type of information to be retained over an extended period of time.

Implicit sequence learning and retention has been widely studied in the motor domain, using the Serial Reaction Time Task (SRTT; Nissen and Bullemer, 1987). In standard versions of the task, a visual stimulus appears in one of four locations on a computer display. Participants are tasked with pressing the corresponding button on a response box that matches the stimulus’ location. Unbeknownst to participants, on some blocks of trials the order that the visual stimulus appears follows a pre-determined sequence. Importantly, in a control block of trials the visual stimuli appear in random order. The typical result observed in healthy adults is that reaction times (RT) decrease as “sequence” blocks are completed and increases during “random” block/s (e.g., Lum et al., 2019). This increase in RT during a random block is typically interpreted as indicating knowledge about the sequence has been acquired, and was affecting manual responses (Robertson, 2007). The decrease in RT across sequence blocks, and increase on the random block, can occur even though the participants may not be able to recall the sequence. Thus, the learning that takes place on the SRTT can be said to be implicit (Schwarb and Schumacher, 2012).

The evidence suggests implicitly acquired sequence knowledge can be retained beyond the initial learning phase. Studies undertaken in this area have found that the sequence on the SRTT can be retained following an interval of minutes (Lum et al., 2018), a day (Ertelt et al., 2012), or a year (Romano et al., 2010). The retention of the sequence has been demonstrated by showing that the difference in RT between the random and sequence blocks during the learning phase is comparable to, or smaller than, the difference between a corresponding set of sequence and random blocks presented at a later time (Wilson et al., 2012; Kemény and Lukács, 2016).

An outstanding question is whether sleep related memory consolidation processes are needed to retain or consolidate an implicitly learnt sequence. Evidence can be found showing sleep promotes the retention of a sequence (Ertelt et al., 2012; Pace-Schott and Spencer, 2013), however, this finding has not always been replicated (Robertson et al., 2004; Kemény and Lukács, 2016). A recent systematic review of this literature by Lerner and Gluck (2019) identified 24 studies investigating the role of sleep in the retention of an implicitly learnt sequence on the SRTT. Of this total, only 10 studies reported sleep-dependent retention effects. The reasons for the inconsistent findings are unclear. To address this issue, the current study examined whether the statistical structure of the sequence determines whether sleep enhances retention.

**FIGURE 1:**
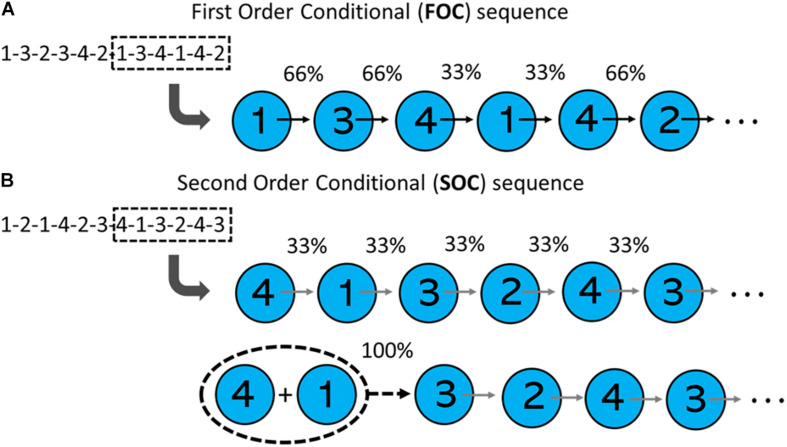
Transitional probabilities of first-order conditional (FOC) and second-order conditional (SOC) sequences. **(A)** Shows that in a FOC sequence (from Deroost et al., 2010), first-order transitions are predictive of element locations. **(B)** Shows that in a SOC sequence (from Smith and McDowall, 2004), the probability of all first-order transitions in a sequence are equal. However, consecutive pairs of elements are predictive of element locations.

In the SRTT literature, a distinction is made between first and second/higher order conditional sequences. An overview of these two sequence types is summarized in Figure 1. In a first order conditional (FOC) sequence, each element in the sequence predicts the next, with varying degrees of probability. For example, in the FOC sequence 1-3-2-3-4-2-1-3-4-1-4-2 (Deroost et al., 2010), if a visual stimulus appears in Position 1, there is a 66% probability that the next stimulus will appear in Position 3, a 33% probability the next stimulus will appear at Position 4, and 0% probability the next stimulus will appear at Position 2. The statistical structure of a second order conditional (SOC) sequence differs. In a SOC sequence, first order transitions are not predictive. Rather, it is the combination of two consecutive elements that is predictive of the following element’s position. For example, in the SOC sequence 1-2-1-3-4-2-3-1-4-3-2-4 (Deroost et al., 2010), Position 1 can be followed by Positions 2, 3, or 4 with equal probability (i.e., 33%). However, the combination of Position 1 followed by Position 3, predicts the next position (Position 4) with 100% probability.

It has been proposed that the learning and retention of SOC sequences may, in part, be supported by the learning and memory functions of the medial temporal lobes (i.e., declarative memory), in addition to the basal ganglia (Poldrack and Rodriguez, 2003; Schendan et al., 2003; Robertson, 2007). However, FOC sequences may be primarily dependent on the basal ganglia. The claim is that in order to learn SOC sequences, the declarative memory system is needed to learn and chunk first order element pairs, which have arbitrary associations. There is some evidence that supports this claim. A number of studies have observed dissociations in FOC and SOC implicit sequence learning in clinical groups that have either medial temporal lobe or basal ganglia abnormalities (Curran, 1997; Smith and McDowall, 2004; Lum et al., 2013; Clark and Lum, 2017). Curran (1997) examined FOC and SOC sequences in participants with temporal lobe damage and healthy controls. One analysis indicated that the healthy controls learnt SOC and FOC sequences with equal proficiency. However, the amnesic group showed poorer learning on the SOC sequence, relative to the FOC sequence. There is also evidence that individuals with Parkinson’s disease, who have basal ganglia pathology, are more proficient learning a SOC sequence relative to a FOC sequence (Smith and McDowall, 2004). Furthermore, non-invasive brain stimulation undertaken with healthy controls has also revealed dissociations in FOC and SOC sequence learning and retention (Lum et al., 2018; Clark et al., 2019). Lum et al. (2018) administered transcranial direct current stimulation over the left prefrontal cortex and temporal lobe, as healthy participants implicitly learnt either a FOC or SOC sequence. The stimulation did not affect the learning or retention of a FOC sequence. However, the stimulation was found to significantly improve retention of the SOC sequence.

Sleep may selectively enhance the retention of an implicitly learnt SOC sequence (but not FOC sequence) via its effects on declarative memory consolidation. Sleep has been found to promote the long-term storage of episodic and semantic information, processed by the medial temporal lobe supported declarative memory system (Marshall and Born, 2007). For example, the ability to remember a list of words can be enhanced if a period of overnight sleep separates the learning and subsequent recall of the words (Plihal and Born, 1997; Wilson et al., 2012). Indeed, even a daytime nap has been found to improve performance on declarative memory tests (Schabus et al., 2005; Lahl et al., 2008). These findings have been attributed to the neural oscillatory dynamics that take place during sleep.

The evidence suggests that brain activity during non-rapid eye movement (NREM) sleep, and especially stage N3 (i.e., slow wave) sleep, plays a role in retaining information processed by the declarative memory system (Rasch and Born, 2013). The electroencephalogram (EEG) of the N3 sleep stage is characterized by low frequency and high amplitude oscillations. This slow wave (i.e., < 4 Hz) activity is thought to promote the retention of information learnt or processed by the hippocampus (Walker, 2009; Born, 2010). It is believed that this low frequency, high power neural oscillatory activity synchronizes brain activity, while simultaneously reactivating recently learnt memory traces residing within the hippocampus. One outcome of this process is that information represented in the hippocampus is redistributed and, over time, becomes represented in the neocortex (Diekelmann and Born, 2010). An increasing neocortical representation renders the information robust to catastrophic interference (i.e., new information overwriting previously acquired information) and is more likely to be retrieved at a future point in time. Consistent with this position, research has shown that an increased amount of time spent in NREM sleep is associated with superior retention on declarative memory tests (Schabus et al., 2005; Tucker and Fishbein, 2008; Wilhelm et al., 2011; Alger et al., 2012).

Memory consolidation processes related to N3 sleep may affect the retention of implicitly learnt SOC sequences, but not FOC sequences. There is some evidence in the SRTT-sleep literature linking sleep to enhanced retention. Several studies that have presented SOC sequences to participants reported enhanced retention following sleep (Robertson et al., 2005; Ertelt et al., 2012; Pace-Schott and Spencer, 2013; Cousins et al., 2014). However, whether these enhancement effects were specifically tied to N3 sleep has yet to be tested. Additionally, studies that examined the retention of a FOC sequence have not found sleep enhancement effects (Spencer et al., 2007; Song and Cohen, 2014). In sum, the extent to which sleep benefits the retention of an implicitly learnt sequence may depend on its statistical structure.

The aim of this study was to examine the effects of sleep on the retention of FOC and SOC sequences. Participants were presented with a SRTT that was administered before and after a 90-min afternoon nap. Half of the participants were presented with a FOC sequence and the other half were presented with a SOC sequence. To obtain baseline levels of learning and retention, participants also completed the SRTT before and after an equivalent duration of daytime wakefulness. The first hypothesis tested in this study was that sleep would benefit the retention of a SOC sequence, but not of a FOC sequence. The second hypothesis tested was that retention of a SOC sequence across sleep would be associated with the time spent in N3 sleep.

## Materials and Methods

### Participants

A total of 40 healthy adults aged between 18 and 39 years of age (*M* = 24.4, *SD* = 3.9) took part in the study. The data from a further three participants were excluded due to technical problems with sleep recording (*n* = 2) or administering the SRTT (*n* = 1). Participants were randomly assigned to be presented with either a FOC sequence (hereafter referred to as the “FOC” group) or SOC sequence (hereafter referred to as the “SOC” group) on the SRTT. None of the participants had previously been diagnosed with a neurological, psychiatric or sleep disorder. All participants self-reported that in the 4 weeks leading up to each session they (a) had an average sleep duration between 6 and 10 h, (b) had not performed overnight shift-work, and (c) that they were not taking any medication/s which affect sleep. All volunteers provided informed consent before taking part in the study and were reimbursed for their time. The project was conducted in accordance with the Declaration of Helsinki and approved by the Deakin University Human Research Ethics Committee.

### Materials

#### Serial Reaction Time Task (SRTT)

Participants completed the SRTT (Nissen and Bullemer, 1987) in two test sessions. In one session, participants completed the task before and after a 90-min afternoon nap session (this session is hereafter referred to as the “Nap” session). In another session, the task was completed before and after a period of sustained wakefulness (this session is hereafter referred to as the “Wake” session). On average, each session was separated by a 15-day interval (FOC Group: *M* = 15.86 days, *SD* = 14.67; SOC Group: *M* = 19.43 days, *SD* = 23.19).

**FIGURE 2:**
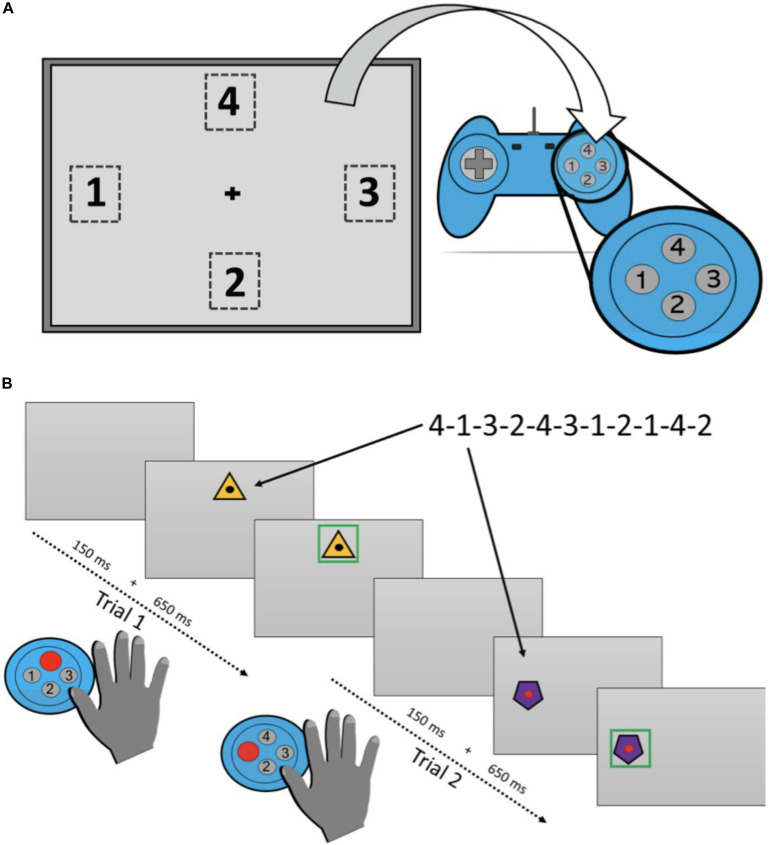
Overview of the Serial Reaction Time Task used in the study. **(A)** Shows the location that the visual stimulus could appear on each trial and the response panel used. **(B)** Provides timing details on a single trial.

The SRTT was divided into a learning phase and a retention phase. A period of approximately 2-h separated each phase of the task. The learning phase was presented before a delay period of napping or wakefulness, depending on the session type, followed by the retention phase. The learning phase comprised six blocks of trials, hereafter labeled Blocks 1–6, respectively. A single trial commenced with a blank computer display (“22” Lenovo ThinkVision T2254pC monitor) for a duration of 150 ms. Next, a visual stimulus appeared for 650 ms in one of four predefined spatial locations presented in a diamond shape around the center of the monitor. Participants were provided with a response pad (Logitech Precision Wired PC Gamepad; Logitech, Lausanne, Switzerland) with four buttons that were also arranged in a diamond configuration. Participants were instructed to press the button on the response pad that matched the location of the visual stimulus. The visual stimulus appeared on the computer display for 650 ms irrespective of whether a response was made. An overview of the task is presented in Figure 2.

Unbeknownst to participants, on Blocks 2, 3, 4, and 5, the location that the visual stimulus appeared on each trial followed a pre-determined sequence. These blocks are hereafter referred to as “Sequence Blocks.” Block 1 was a practice block which served to familiarize participants with the task and consisted of 60 trials. On this block the visual stimulus appeared randomly in one of the four locations. All other blocks consisted of 96 trials. Block 6 was the “Random Block.” On this block, the visual stimulus appeared pseudo-randomly in one of the four spatial locations adhering to the constraints that they (a) always began at location “1” and (b) did not contain five or more stimuli locations which matched the sequence used in that session.

Participants were presented with the same type of sequence (i.e., FOC or SOC sequence) in both the sleep and wake sessions. However, a different FOC and SOC sequence was used in each of the sessions. Thus, in total, four different sequences were used in this study. Participants were presented with two FOC sequences (one in the Nap session and the other in the Wake session) or two SOC sequences (one in the Nap session and the other in the Wake session). Labeling the left-most position that the visual stimulus could appear on the screen as “1,” the lower most position as “2,” and so on, the sequences presented to participants were as follows:

• FOC sequences:
  • 1-3-2-3-4-2-1-3-4-1-4-2 (from Deroost et al., 2010)
  • 1-4-1-3-4-2-3-2-1-3-4-2 (from Smith and McDowall, 2004)
• SOC sequences:
  • 1-2-1-3-4-2-3-1-4-3-2-4 (from Deroost et al., 2010)
  • 1-2-1-4-2-3-4-1-3-2-4-3 (from Smith and McDowall, 2004)

The presentation order of the different FOC and SOC sequences within each group was randomized by a coin-toss before commencing the learning phase of the SRTT on the first session.

The retention phase of the SRTT comprised three blocks of 96 trials. This part of the task was administered after the nap or wakeful period. Blocks 7 and 9 were the Sequence blocks. On these blocks, participants were presented with the same FOC or SOC sequence used on the learning phase of the task. Block 8 comprised the Random block in which the visual stimulus appeared in pseudo-random positions using the same constraints described above as for Block 6 (i.e., random block in the learning phase).

The SRTT was presented, and responses recorded, using E-Prime 2.0 software. Both accuracy and reaction times were recorded as participants completed the Learning and Retention phase of the task. Trials in which participants did not make a response were coded as incorrect. For each participant, the mean RT for each block was computed. These data were used to examine learning and retention of the FOC and SOC sequences.

In the SRTT literature, RT are the main dependent variable of interest, however, accuracy data were analyzed to ensure participants were correctly responding to the visual stimulus. Table 1 presents the mean proportion of correct responses reported by FOC/SOC learning group and Nap/Wake session. This table shows that accuracy approached ceiling for both groups (i.e., FOC and SOC groups), and test sessions (i.e., Nap, Wake session). To formally test for differences in accuracy, the proportion of correct responses were submitted to a 2 (Group: FOC, SOC) × 2 (Session: Nap, Wake) × 9 (Block: Block 1–9) Mixed Design Factorial ANOVA. To correct for non-normality an arcsine transformation was applied to the data. Non-significant differences and small effect sizes were observed for the main effects of Group [*F*_(1, 38)_ = 0.652, *p* = 0.425, η*_*p*_*^2^ = 0.017] and Session [*F*_(1, 38)_ = 0.761, *p* = 0.389, η*_*p*_*^2^ = 0.020] on accuracy. A significant effect of Block on accuracy was observed [*F*_(8, 304)_ = 10.773, *p* < 0.001, η*_*p*_*^2^ = 0.221]. The main effect of Block can be attributed to accuracy being slightly lower in Block 6 relative to the other blocks (see Supplementary Material Analysis for pairwise comparisons of means). All interaction terms were non-significant and associated with small effect sizes {Group × Session: [*F*_(1, 38)_ = 0.102, *p* = 0.751, η*_*p*_*^2^ = 0.003]; Group × Block: [*F*_(1, 38)_ = 0.996, *p* = 0.439, η*_*p*_*^2^ = 0.026]; Group × Session × Block: [*F*_(1, 38)_ = 0.176, *p* = 0.994, η*_*p*_*^2^ = 0.005]}. These analyses indicate that, overall, there was no evidence to suggest a difference in accuracy between the FOC/SOC Groups and Nap/Wake Sessions.

**TABLE 1:** Proportion of correct responses on the SRTT reported by FOC/SOC sequence learning groups and nap/wake conditions.

Block number	FOC group				SOC group			
	Nap session		Wake session		Nap session		Wake session	
	*M*	*SD*	*M*	*SD*	*M*	*SD*	*M*	*SD*
B1	0.97	0.05	0.98	0.04	0.96	0.05	0.97	0.04
B2	0.97	0.05	0.98	0.05	0.98	0.02	0.98	0.03
B3	0.97	0.04	0.98	0.04	0.97	0.03	0.97	0.04
B4	0.96	0.05	0.97	0.04	0.96	0.07	0.97	0.04
B5	0.97	0.05	0.97	0.06	0.96	0.04	0.96	0.04
B6	0.95	0.07	0.95	0.07	0.94	0.05	0.96	0.04
B7	0.98	0.03	0.98	0.04	0.97	0.02	0.97	0.03
B8	0.98	0.05	0.98	0.04	0.98	0.02	0.97	0.04
B9	0.98	0.03	0.98	0.03	0.98	0.02	0.98	0.02

### Measure of Sleepiness Before and After Each Nap/Wake Session

Differences between the group’s vigilance, both before and after the nap/wakefulness period may potentially influence their performance on the SRTT (Glenville et al., 1978). The “Karolinska Sleepiness Scale” (KSS; Åkerstedt and Gillberg, 1990) was used to measure participant sleepiness before they completed the learning phase on the task, and after they completed the retention phase. This scale comprises 10 items in which participants rate their feeling of sleepiness on a 9-point scale (1 = “Extremely Alert” to 10 = “Extremely sleepy, can’t keep awake”). Higher scores indicate greater levels of sleepiness at the time of completing the questionnaire.

#### Background Measures

Participants were also presented with a series of tasks and surveys to measure handedness and general cognitive functioning. Data from these tasks were obtained to evaluate whether participants in the FOC and SOC groups differed on variables that might explain potential group differences in sequence retention.

Handedness was measured using the Edinburgh Handedness Inventory (Oldfield, 1971). This inventory comprises 12 items in which participants indicate which hand/foot/eye is used to complete common tasks. The inventory was scored on a scale of −100 to + 100, where positive values indicate a tendency to be right-handed, and negative values indicate being left-handed. To obtain an estimate of general cognitive functioning, participants were administered the Matrix Reasoning subtest from the Weschler Abbreviated Scale of Intelligence (Weschler, 1999). The Matrix Reasoning subtest was used to measure non-verbal reasoning. Performance on this subtest is described as a T-score, standardized to a mean of 50 and standard deviation of 10. Table 2 presents summary data for each of these instruments along with results from statistical tests comparing the two groups on each measure. Summary data reporting the age and number of females in each group are also reported in Table 2. For all variables, non-significant differences between groups were observed.

**TABLE 2:** Descriptive characteristics of FOC and SOC sequence learning groups.

	FOC		SOC			
Variable	*M*	*SD*	*M*	*SD*	*t* or χ^2^	*p*-value
Age (years)	23.3	4.4	25.40	3.5	1.661	0.105
Gender (% female)	90%	−	60%	−	3.584	0.058
Edinburgh handedness inventory	66.4	58.8	75.2	46.8	0.456	0.604
Matrix reasoning subtest	55.0	6.7	57.7	6.6	1.346	0.186

### Procedure

**FIGURE 3:**
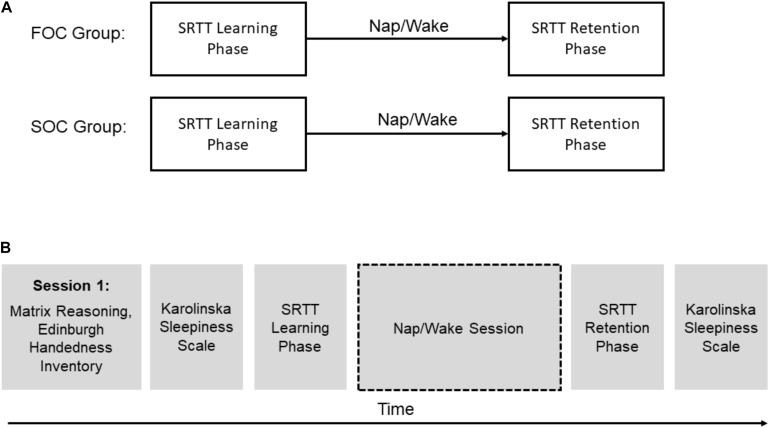
An overview of the study’s design. **(A)** Shows presentation of the learning and retention phases of the Serial Reaction Time Task for FOC and SOC groups. **(B)** Presents a timeline showing the order surveys, standardized tests and the Serial Reaction Time Task was administered for all participants.

Participants in both groups completed two sessions. Figure 3 presents an overview of the testing protocol. In one of the sessions, participants completed the learning and retention phases of the SRTT, separated by a 2-h delay containing a 90-min daytime nap (i.e., “Nap” session). In a control condition, the learning and retention phases of the SRTT were separated by a period of wakefulness (i.e., “Wake” session). During the Wake session, participants were seated upright in a chair and watched a film which contained only musical accompaniment (“Baraka” or “Samsara”). During both sessions polysomnography (PSG) was acquired (details provided below). In the Nap session, this was to determine the time spent in each sleep stage. In the Wake session, this was used to verify that participants did not fall asleep. Before each session, participants were requested to adhere to their regular sleep-wake cycle for a week leading up to each session, but were requested to wake up 1-h earlier than their usual waking time on the day of the experiment. Adherence to these requests was verified by examining a self-reported, 7-day sleep diary completed by the participants.

During the first session, participants completed the Matrix Reasoning subtest, Edinburgh Handedness Inventory, and Karolinska Sleepiness Scale. Participants then completed the learning phase of the SRTT followed by a 2-h delay period, during which the participants slept or watched the documentary. In both Nap and Wake sessions, the end of the delay period included a 20-min break to allow participants to recover before the second administration of the SRTT. Following this, the retention phase of the SRTT was administered and then participants completed the second of the two presentations of the Karolinska Sleepiness Scale. During the second session, participants also completed the Karolinska Sleepiness Scale before the learning phase and after the retention phase of the SRTT.

### Polysomnography Recording

EEG was continuously acquired as participants slept (as part of the Nap session) or watched the film (as part of the Wake session). EEG was acquired using a SynAmps RT recording system running Curry 7 software (NeuroScan, Compumedics) via 12 Ag/AgCl electrodes placed in positions AFz, F7, Fz, F8, C3, Cz, C4, P3, Pz, P4, O1, and O2. Two additional electrodes were placed on the left and right mastoid. During the recording, EEG was grounded at FPz and referenced to FCz. Also, two electrooculography (EOG) and three chin electromyography (EMG) electrodes were measured to allow sleep scoring as per the recommendations of the AASM (Iber et al., 2007). EEG electrode impedances were reduced to < 10 kΩ at the beginning of data acquisition. Online visualization of participants’ sleeping EEG was performed with scalp electrodes re-referenced to the mastoids, and data filtered using a 0.5–60 Hz band-pass filter coupled with a 50 Hz notch filter. Online polysomnography scoring was also performed to monitor for the presence of participant sleepiness during the Wake control sessions. Using this data, it was confirmed none of the participants in the Wake session entered N2, N3 or REM sleep stages. EEG, EOG, and EMG data for both sessions were also recorded and stored for offline analysis. All data were recorded at a sampling rate of 1,000 Hz, referenced to FCz and without acquisition filters.

### Sleep Stage Scoring

EEG data were pre-processed and analyzed using EEGLAB 14.1.2b (Delorme and Makeig, 2004) and ERPLAB 5.0 (Lopez-Calderon and Luck, 2014) run in MATLAB 2017 (MathWorks, Natick, MA, United States). Before sleep scoring, EEG were down sampled to 250 Hz, notch filtered at 50 Hz and band-pass filtered between 0.3 and 60 Hz. Each 30 s epoch of the Nap and Wake session was visually scored independently by two researchers (MB, LF) according to standard AASM guidelines (Iber et al., 2007). Each epoch was categorized as N1, N2, N3, REM, or wake. Disagreements in sleep scoring were reconciled by a third scorer (RC). Stages N1, N2, and N3 were classified together as NREM sleep. Stage N3 sleep constituted “slow-wave sleep” (SWS). The Time in Bed (TIB) and total sleep time (TST) was also computed. All variables were calculated in minutes.

## Results

The dataset from this study is publicly available for download from the Open Science Framework platform^1^.

### Measure of Sleepiness and Time Spent in Each Sleep Stage

The first set of analyses compared the FOC and SOC groups with respect to scores from the Karolinska Sleepiness Scale, which was administered before and after both phases of the SRTT were completed. These analyses tested whether there were significant differences between the groups with respect to levels of sleepiness. Summary data from this instrument reported by group (i.e., FOC or SOC group) and session (i.e., Nap or Wake) are presented in Table 3. These data were submitted to a 2 (Group: FOC, SOC) × 2 (Session: Nap, Wake) × 2 (Time: Pre-Nap/Wakeful Period, Post-Nap/Wakeful Period) Mixed Design Factorial ANOVA. The only significant result to emerge from this analysis was the interaction between Session and Time [*F*_(1, 38)_ = 20.31, *p* < 0.001, η*_*p*_*^2^ = 0.290]. This interaction arose because participants, in both FOC and SOC groups, reported feeling less sleepy after a nap, compared to the wakeful period. All main effects were non-significant [Session: [*F*_(1, 38)_ = 1.28, *p* = 0.265, η*_*p*_*^2^ = 0.033]; Time: [*F*_(1, 38)_ = 2.69, *p* = 0.109, η*_*p*_*^2^ = 0.066]; Group: [*F*_(1, 38)_ = 1.80, *p* = 0.187, η*_*p*_*^2^ = 0.045]]. All interaction terms were also non-significant [Session × Group: [*F*_(1, 38)_ = 0.14, *p* = 0.708, η*_*p*_*^2^ = 0.004]; Time × Group: [*F*_(1, 38)_ = 1.69, *p* = 0.201, η*_*p*_*^2^ = 0.043]; Session × Time × Group: [*F*_(1, 38)_ = 2.11, *p* = 0.155, η*_*p*_*^2^ = 0.053]]. These analyses indicate no significant differences in sleepiness between the FOC and SOC groups.

**TABLE 3:** Summary statistics reporting subjective sleepiness from the Karolinska Sleepiness Scale reported by group (FOC, SOC) and session (nap, wake).

Session	FOC group		SOC group	
	*M*	*SD*	*M*	*SD*
**Nap**				
Pre-nap period	6.2	1.1	6.6	1.5
Post-nap period	5.1	1.7	5.5	1.9
**Wake**				
Pre-wakefulness period	5.4	1.4	5.4	1.6
Post-wakefulness period	5.2	1.8	6.3	1.7

The next set of analyses tested whether the FOC and SOC groups differed with respect to the amount of time spent in the studied sleep stages. Table 4 presents summary data reporting the total time in bed, total wake time, total sleep time and amount of time in N1, N2, N3, and REM sleep stages, reported by FOC/SOC group during the Nap session. All variables are reported in minutes. Also presented in Table 4 is the mean proportion of time participants spent in SWS. Results from independent samples *t*-tests revealed no significant differences between groups on these variables. Results from these analyses are also presented in Table 4. Crucially, with respect to time in N3 sleep, the groups were statistically indistinguishable. Overall, these analyses indicate that the two groups were not found to be statistically different with respect to the time spent in each of the examined sleep stages during the Nap session.

**TABLE 4:** Sleep architecture of 90-min Nap for FOC and SOC groups.

Outcome variable	FOC group		SOC group		*t-*value^*a*^	*p*-value	Cohen’s *d*^*a*^
	*M*	*SD*	*M*	*SD*			
Time in bed (min)	89.5	0.6	89.9	1.5	1.150	0.257	0.364
Total wake time (min)	18.7	25.8	22.6	17.5	0.555	0.582	0.176
Total sleep time (min)	70.3	25.7	66.6	17.5	−0.540	0.592	−0.171
Proportion time in N3 (%)	20.6%	21.1%	19.2%	17.5%	−0.236	0.814	−0.075
N1 (min)	15.5	16.3	11.5	6.0	−1.016	0.316	−0.321
N2 (min)	31.9	17.7	33.0	13.3	0.232	0.818	0.073
N3 (min)	16.5	18.0	13.7	12.4	−0.577	0.567	−0.183
REM (min)	6.5	12.1	8.4	9.8	0.539	0.593	0.170

*^a^Positive values indicate SOC group had longer duration.*

### Analyses Examining Learning and Retention of FOC and SOC Sequences

**FIGURE 4:**
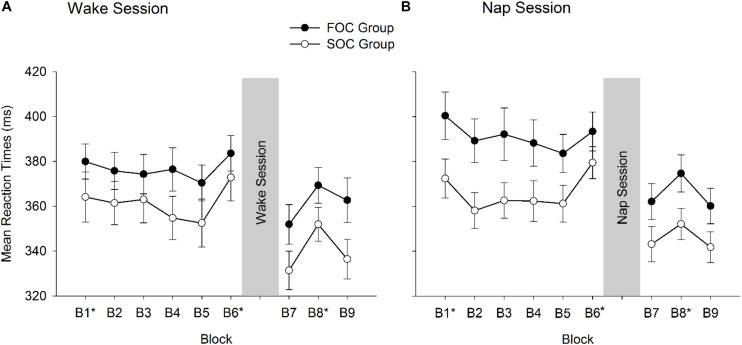
Mean reaction times from the Serial Reaction Time Task reported by FOC and SOC groups and Block. **(A)** Shows data from the Wake session. **(B)** Shows data from the Nap session. Blocks 1–6 correspond to the Learning phase of the task. Blocks 7–9 correspond to the Retention phase of the task. Blocks marked with * indicate the random blocks. Error bars show standard error.

Analyses from the SRTT are now presented. Figure 4 presents mean RT reported by Block (1, 2, 3, 4, 5, 6, 7, 8, 9), Phase of Task (Learning, Retention), Group (FOC Group, SOC Group) and Session Type (Nap, Wake). Figure 4A presents RT data for the FOC and SOC groups in the Wake session, and Panel B presents data from FOC and SOC groups in the Nap session. Preliminary analyses of the SRTT data examined whether both sequence types were learnt and retained. In the Learning phase differences in RT were compared between Block 5 (sequence block) and Block 6 (random block). In the retention phase, differences in RT were compared between the average of Blocks 7 and 9 (sequence blocks) and Block 8 (random block). As noted earlier, an increase in RT from the sequence block to the random block is typically used to determine whether sequence knowledge was influencing manual responses (Robertson, 2007). Thus, if participants had learnt and/or retained the sequence, RT should be significantly larger in the random block compared to the sequence block/s. This was tested using paired samples *t*-tests with Bonferroni corrected *p*-values. For the FOC group, paired samples *t*-tests indicated that in both Nap and Wake sessions, RT significantly increased in the random block, compared to the sequence block/s, in the learning (Nap: *p* = 0.028; Wake: *p* = 0.004) and retention phases (Nap: *p* = 0.002; Wake: *p* = 0.004). This was also the case for the SOC group. RT were significantly slower in the random block compared to sequence blocks in both learning (Nap: *p* = 0.002; Wake: *p* = 0.006) and retention phases (Nap: *p* = 0.032; Wake: *p* = 0.002) in the Wake and Nap sessions. Collectively, these results indicate that for both FOC and SOC groups, the sequence was learnt and then retained, following either a nap or period of wakefulness.

**FIGURE 5:**
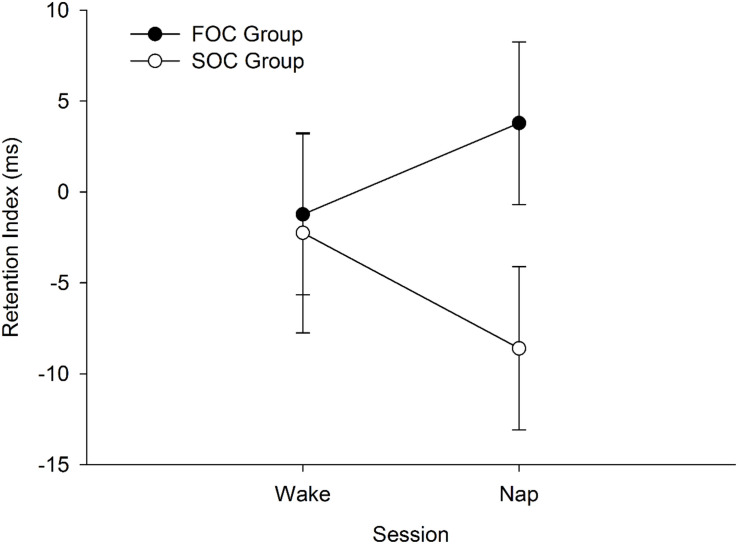
Mean Sequence Retention Index reported by FOC/SOC Group and Session (i.e., Wake, Nap). Positive values indicate enhanced retention of the sequence. Error bars show standard error.

A composite variable was created to test the hypothesis that sleep would preferentially enhance retention of a SOC sequence relative to a FOC sequence. For each participant, a “Sequence Retention Index” was created that took into account performance on the learning phase. This was achieved by first computing the difference between the final sequence and random blocks on the learning and retention phases of the SRTT. This calculation was undertaken so that positive values indicate RT were slower on the random block, compared to the preceding sequence block. The difference score from the wake session was subtracted from the corresponding value in the nap session, that is: [(Block 8_random block retention phase_ – Average of Blocks 7 and 9_sequence blocks retention phase_)—(Block 6_random block learning phase_ – Block 5_sequence block learning phase_)]. Positive values on the Retention Index would indicate that there was a stronger sequence knowledge effect in the retention phase compared to learning phase. Negative values would indicate “forgetting” of the sequence. The Retention Index was computed separately for Nap and Wake sessions. Figure 5 presents the mean Sequence Retention Index reported by Group (FOC Group, SOC Group) and Session (Nap, Wake).

The Sequence Retention Index was submitted to a 2 (Group: FOC, SOC) × 2 (Session: Nap, Wake) Mixed Design Factorial ANOVA. The main effects of Session [*F*_(1, 38)_ = 0.02, *p* = 0.899, η*_*p*_*^2^ < 0.001] and Group [*F*_(1, 38)_ = 2.44, *p* = 0.127, η*_*p*_*^2^ = 0.060] were not significant. The interaction between these two terms was also not significant [*F*_(1, 38)_ = 1.22, *p* = 0.277, η*_*p*_*^2^ = 0.031]. The non-significant interaction indicates that sleep did not preferentially enhance retention of the SOC sequence, relative to the FOC sequence.

A separate composite value was created to test whether non-sequence related general motor learning was improved following sleep and/or wakefulness for either the FOC or SOC groups. To achieve this, a “Motor Learning Index” was computed for each participant comparing performance on the final sequence block of the learning phase of the task with performance on the first sequence block of the retention phase of the task. Specifically, the Motor Learning Index was calculated by subtracting RTs on Block 5 from RTs on Block 7; that is, [Block 5 sequence block learning phase − Block 7 sequence block retention phase]. Positive values indicate an increase in motor speed following the delay relative to motor speed on the final sequence block at the end of the training phase. Negative values indicate decreases in motor speed following the delay relative to the end of the training phase.

The Motor Learning Index was submitted to a 2 (Group: FOC, SOC) × 2 (Session: Nap, Wake) Mixed Design Factorial ANOVA. Both the main effect of Session [*F*_(1, 38)_ < 0.001, *p* = 0.997, η*_*p*_*^2^ < 0.001] and Group [*F*_(1,38)_ = 0.004, *p* = 0.950, η*_*p*_*^2^ < 0.001] were not significant. The interaction between these two terms was also not significant [*F*_(1, 38)_ = 0.38, *p* = 0.542, η*_*p*_*^2^ = 0.004]. The non-significant main and interaction effects indicate sleep did not enhance general motor learning more than a period of waking for either the FOC or SOC sequence learning groups.

### Relationship Between Time Spent in Sleep Stages With Sequence Retention and Motor Learning

The final set of analyses examined the association between the time spent in each sleep stage, with the Sequence Retention Index and the Motor Learning Index. Correlations between these variables were computed separately for the FOC and SOC groups. These analyses were undertaken to test the hypothesis that retention of a SOC sequence would be correlated with the time spent in N3 sleep. That is, participants who spent more time in N3 or slow wave sleep, would evidence superior retention of the SOC sequence. Results from these correlation analyses are presented in Table 5. No statistically significant correlations were found between sequence retention or general motor learning with the time spent in the different sleep stages.

**TABLE 5:** Pearson’s correlations between the Sequence Retention Index, the Motor Learning Index and time spent (in min) in each sleep stage.

	Sequence retention index				Motor learning index			
Sleep stage	FOC group		SOC group		FOC group		SOC group	
	*r*	*p*-value	*r*	*p*-value	*r*	*p*-value	*r*	*p*-value
N1	0.129	0.588	0.393	0.087	−0.117	0.624	−0.040	0.865
N2	−0.132	0.578	0.022	0.962	0.116	0.626	0.226	0.339
N3	0.189	0.424	0.050	0.834	0.213	0.367	−0.335	0.149
REM	−0.288	0.219	0.073	0.760	−0.050	0.833	0.133	0.575

*Correlations reported separately for FOC and SOC Groups.*

## Discussion

This study examined the effect of sleep on the retention of implicitly learnt FOC and SOC sequences. The results from the study did not support the hypothesis that sleep would enhance retention of a SOC sequence. The analyses revealed both FOC and SOC sequences were equally well retained across a delay period comprising either a nap or a period of wakefulness. Also, the amount of time spent in N3 sleep (i.e., slow wave sleep) was not related to the retention of the SOC sequence. These findings question whether the declarative memory system is needed to store implicitly learnt SOC sequences (Poldrack and Rodriguez, 2003; Schendan et al., 2003; Robertson, 2007). Also, at a more general level, the study provides evidence showing that a daytime nap conferred no benefit to the retention of an implicitly learnt motor sequence over a 2-h period.

As noted earlier, 10 out of 24 studies reviewed by Lerner and Gluck (2019) found that sleep enhanced the retention of an implicitly learnt sequence on the SRTT. Several of these studies that found sleep promoted retention had presented second- or higher- order sequences to participants (Robertson et al., 2005; Ertelt et al., 2012; Pace-Schott and Spencer, 2013; Cousins et al., 2014). Our results indicate that the statistical structure of the sequence used on the SRTT does not explain inconsistencies in this literature. A commonly used method to determine whether a sequence has been learnt and retained on the SRTT is to compare RT on sequence and random blocks (e.g., Meier and Cock, 2014). Our results showed a significant increase in RT from sequence to random blocks on both the learning and retention phases. This result was observed when participants were presented with a FOC and SOC sequence, and in both nap and wake conditions. These analyses demonstrate that participants learnt and retained both sequence types following a nap, or period of wakefulness.

Critically, the analyses examining whether sleep was related to the retention of the SOC sequences were all non-significant. For these analyses, insufficient statistical power to detect a meaningful effect does not appear to explain our results. A small-to-medium effect size (η*_*p*_*^2^) was associated with the interaction term testing whether the Sequence Retention Index was higher for the SOC group, relative to the FOC group, following a nap. Moreover, a trend is present in Figure 5 indicating that sleep had a negative impact on the retention of a SOC sequence. This was not the case for the FOC sequence, in which there is a trend indicating sleep improved retention of this sequence type. These results were not in the predicted direction. Also, the correlation coefficient measuring the time spent in N3 and the retention of the SOC sequence was small in magnitude (*r* = 0.050). These findings show that sleep has no discernible effect on the retention of a SOC (or FOC) sequence, at least following a daytime nap. This finding contrasts with research undertaken with list-learning tasks which are known to depend on the declarative memory system. In this literature, a daytime nap has been found to enhance retention of this type of information (Tucker and Fishbein, 2008).

One interpretation of these results may be that the retention of FOC and SOC sequences is supported by the same memory system. Specifically, in line with past meta-analysis of fMRI data, the initial implicit learning of a motor sequence appears to be supported by the basal ganglia (Janacsek et al., 2020). Following 2-24-h after learning, the evidence suggests that motor sequence information is re-represented in several cortical structures, including the primary motor cortex (Muellbacher et al., 2002; Lohse et al., 2014). Representation of the motor sequence in cortical structures appears to be more dependent on time, rather than the presence (or absence) of sleep. Specifically, there is some evidence suggesting that the retention process is supported by high frequency oscillatory activity that is localized in the primary motor cortex, that is not contingent on sleep (for a review see Robertson, 2009). The findings of the current study might therefore be indicating that this type of oscillatory activity, or another common time-dependent retention process, supports the retention of both FOC and SOC sequences.

How might our results relate to findings of a dissociation between learning FOC and SOC sequences? One suggestion is that there may be subtle differences in the neural regions responsible for learning, but not retention, of FOC and SOC sequences. The engagement of neural regions (in addition to the basal ganglia) appears to change across the task (Seidler et al., 2005; Rieckmann et al., 2010; Kupferschmidt et al., 2017). For example, Rieckmann et al. (2010) found that participants learning a SOC sequence showed activation of medial temporal lobes during the early learning stages of the SRTT, but this activation decreased across the task. It may be that, if hippocampal involvement is required to track the ambiguous transitions of a SOC sequence, this involvement is early and transitory. The later learning stages, and the retention processes, may be based in the basal ganglia and motor cortices for both SOC and FOC sequences.

Involvement of the declarative memory system on the SRTT may still explain results from past studies, that found sleep promoted the retention of an implicitly learnt sequence. However, the involvement of this memory system may be linked to general learning conditions, rather than the statistical structure of the sequence. A number of studies that have found sleep enhances retention of a SOC sequence, have used contextual cueing variants of the SRTT (e.g., Cousins et al., 2014, 2016). In these studies, a cue is presented during the learning phase of the task. Depending on the study, the cue may be a tone (e.g., Cousins et al., 2014) or olfactory stimulus (e.g., Diekelmann et al., 2016). During the retention phase, the cue is then re-introduced. Contextual cueing may introduce an episodic memory component to the SRTT. Since episodic memory is supported by the declarative memory system (Burgess et al., 2002), contextual cueing may bias the sequence to being processed by the hippocampus. Once the sequence is represented in this structure, retention may be more likely to be promoted via the effects of slow wave sleep, as appears to be the case for other information processed by this memory system (Born, 2010). Thus, it is the general learning condition, rather than sequence structure, that may influence the extent the declarative memory system is involved in the retention of an implicitly learnt motor sequence.

This study is not without limitations. First, the present findings relate to the use of a short duration nap and as such does not exclude the possibility that a longer overnight period of sleep might benefit implicit sequence retention. There is some evidence which suggests a daytime nap can provide similar benefits to the performance of procedural learning tasks (e.g., Mednick et al., 2003; Doyon et al., 2009). However, van Schalkwijk et al. (2019) showed performance on a procedural motor learning task improved significantly after 7-h of overnight sleep, but not after 90-min of daytime napping. Additional research is therefore needed to examine whether there may be a dose-dependent relationship between the duration of NREM sleep and sequence retention.

A second limitation is that sequence retention might be influenced by the typical napping habits of the participants involved in this study. In this study, participants were not screened regarding their napping behavior outside of the 7-days prior to each study session. This could be important as the effects of sleep on cognition may be influenced by whether participants habitually nap or not (Milner and Cote, 2009). Milner et al. (2006) showed procedural motor task performance was improved after a 20-min nap and was associated with NREM sleep spindle activity, but only in participants who were habitual nappers. Future research should examine whether experience with napping mediates the retention of first-order and second-order conditional sequences.

## Conclusion

This study provides evidence that a daytime nap does not enhance the retention of implicitly learnt FOC or SOC sequences on the SRTT. The absence of sleep-related retention effects for the SOC sequences questions the involvement of the declarative memory system in processing higher order sequences. Moreover, the study suggests that in the short-term (1–2 h), the retention of implicitly learnt sequences may be supported by similar neural processes, irrespective of its statistical structure.

## Data Availability Statement

The datasets presented in this study can be found in OSF at the following link: https://osf.io/7sdcr/?view_only=bec97a609b704681ab41b6b5a5e7f001.

## Ethics Statement

The studies involving human participants were reviewed and approved by the Deakin University Human Research Ethics Committee. The patients/participants provided their written informed consent to participate in this study.

## Author Contributions

MB and JL designed the experiment. MB collected the data. RC provided training on sleeping EEG acquisition and sleep scoring. MB, LF, and RC conducted sleep scoring. MB, JL, and GC conducted the analyses and wrote the manuscript. MB, JL, RC, LF, PE, and GC edited the manuscript. All authors contributed to the article and approved the submitted version.

## Conflict of Interest

The authors declare that the research was conducted in the absence of any commercial or financial relationships that could be construed as a potential conflict of interest.

## Footnotes

1 https://osf.io/7sdcr/?view_only=bec97a609b704681ab41b6b5a5e7f001
