# Supplementary material for: A Daytime Nap Does Not Enhance the Retention of a First-Order or Second-Order Motor Sequence
Source: Front Behav Neurosci. 2021 Jul 16;15:659281. doi: 10.3389/fnbeh.2021.659281 (PMC8324096; doi:10.3389/fnbeh.2021.659281)
Supplement: Supplementary file 1 [file Table_1.DOCX]

Supplementary Analysis

Pairwise Comparison of Means Comparing Differences in Accuracy Between Blocks. Table shows Bonferroni corrected *p*-values.

|  | B1 | B2 | B3 | B4 | B5 | B6 | B7 | B8 | B9 |
| --- | --- | --- | --- | --- | --- | --- | --- | --- | --- |
| B1 | - |  |  |  |  |  |  |  |  |
| B2 | 0.999 | - |  |  |  |  |  |  |  |
| B3 | 0.999 | 0.396 | - |  |  |  |  |  |  |
| B4 | 0.797 | 0.001 | 0.999 | - |  |  |  |  |  |
| B5 | 0.217 | <.001 | 0.999 | 0.999 | - |  |  |  |  |
| B6 | <.001 | <.001 | <.001 | 0.087 | 0.355 | - |  |  |  |
| B7 | 0.999 | 0.704 | 0.999 | 0.999 | 0.661 | <.001 | - |  |  |
| B8 | 0.999 | 0.999 | 0.999 | 0.213 | 0.049 | <.001 | 0.999 | - |  |
| B9 | 0.999 | 0.999 | 0.834 | 0.003 | <.001 | <.001 | 0.999 | 0.999 | - |
